# The complete chloroplast genome of *Arisaema bockii* Engler and its phylogenetic analysis in the family Araceae

**DOI:** 10.1080/23802359.2021.1993107

**Published:** 2021-10-23

**Authors:** Shanyong Yi, Tao Xu, Xiangwen Song, Wei Wang, Guanglin Wang, Wangyang Yu, Bangxing Han

**Affiliations:** a Department of Biological and Pharmaceutical Engineering, West Anhui University, Luʼan, P.R. China; b Anhui Engineering Laboratory for Conservation and Sustainable Utilization of Traditional Chinese Medicine Resources, West Anhui University, Luʼan, P.R. China; c Analytical and Testing Center, West Anhui University, Luʼan, P.R. China; d Anhui Qiansouyan Biotechnology Co., Ltd, Luʼan, P.R. China

**Keywords:** *Arisaema bockii*, complete chloroplast genome, phylogenetic analysis, Araceae

## Abstract

*Arisaema bockii* Engler is a perennial herbaceous medicinal plant, which is widely distributed in many provinces in China such as Anhui, Jiangsu, and Zhejiang. In this study, the complete chloroplast genome sequence of *A. bockii* was assembled and characterized based on high-throughput sequencing data. The total length of chloroplast genome was 175,537 bp, including large single-copy (LSC) and small single-copy (SSC) regions of 98,870 bp and 23,345 bp, respectively, which were separated by a pair of 27,161 bp inverted repeat (IR) regions. The genome contained 129 genes, including 84 protein-coding genes, 36 tRNA genes, 8 rRNA genes, and one pseudogene. The overall GC content of the genome was 33.6%. A phylogenetic tree reconstructed by 30 chloroplast genomes revealed that *A. bockii* was mostly related to the same genus species *A. ringens, A. franchetianum* and *A. erubescens.* The work reported the first complete chloroplast genome of *A. bockii*, which may provide some useful information to the evolution of the family Araceae.

*Arisaema bockii* Engler is a kind of Chinese herbal medicine, which is widely distributed in China and south of Japan. It is one of the most beautiful species in the *Arisaema* genus of the family Araceae (Hayakawa et al. 2011). Its medicinal parts are tubers, which can be used as the substitute for *A. erubescens, A. heterophyllum*, and *A. amurense* listed in Chinese Pharmacopeia with the function of dissipating binds and dispersing swelling (Wang et al. 2012). Pharmacological analysis indicated that *A. bockii* has the activities of anti-tumor (Feng et al. 2016), anti-bacterial (Wang et al. 2012), and nematicidal (Du et al. 2011). Flavonoids, terpenoids, alkaloids and glycosphingolipids are its main medicinal ingredients (Kant et al. 2020). However, little is known about its biosynthesis mechanism of the compounds. In addition, its phylogenetic position is not very clear causing on the lack of genomic information. Here, we characterized the complete chloroplast genome sequence of *A. bockii* using high throughput sequencing technology, which will provide useful informatics data for the phylogeny of *A. bockii* and other related species.

The fresh leaves of *A. bockii* were collected from Lu’an, Anhui, China (31°77′ N, 115°93′ E). Specimens were stored in the Herbarium of West Anhui University (the accession number is WAU-DTL-20210218-1, and the email of the sample contact is ysy345283991@163.com). Total genomic DNA was extracted from the leaves according to a modified CTAB protocol (Doyle and Doyle 1987). The DNA was stored at −80 °C in our lab. The whole genome sequencing was conducted by Hefei Biodata Biotechnologies Inc. (Hefei, China) on the Illumina Hiseq platform. The filtered sequences were assembled by using the program SPAdes assembler 3.10.0 (Bankevich et al. 2012). The chloroplast genes were annotated by GeSeq (Tillich et al. 2017) with default parameters to predict protein-coding genes, rRNA and tRNA genes. All tRNA genes were further verified by using tRNAscan-SE 2.0 (Chan et al. 2021).

The chloroplast genome of *A. bockii* was determined to comprise a 175,537 bp, double stranded, and circular DNA (GenBank accession no. MZ380241), containing two inverted repeat (IR) regions of 27,161 bp, and separated by large single-copy (LSC) and small single-copy (SSC) regions of 98,870 bp and 23,345 bp, respectively. The genome was predicted to have 129 genes, including 84 protein-coding genes, 36 tRNA genes, 8 rRNA genes, and one pseudogene. Five protein-coding genes, seven tRNA genes and four rRNA genes were duplicated in IR regions. Nineteen genes contained two exons and four genes (*clp*P, *ycf*3 and two *rps*12) contained three exons. The overall GC content of *A. bockii* cp genome was 33.6% and the corresponding values in LSC, SSC and IR regions were 31.6%, 27.4% and 39.9%, respectively.

To investigate its taxonomic status, alignment was performed with 30 reported chloroplast genome sequences of the family Araceae (*Centella asiatica* and *Sanicula chinensis* were used as outgroup) using MAFFT v7.307 (Katoh and Standley 2013), and then a maximum likelihood (ML) tree was constructed by FastTree version 2.1.10 with Generalized Time-Reversible (GTR) model, 1000 bootstrap replicates (Price 2010). As expected, *A. bockii* was mostly related to the same genus species *A. ringens, A. franchetianum* and *A. erubescens* with bootstrap support values of 100% (Figure 1).

**Figure 1.**
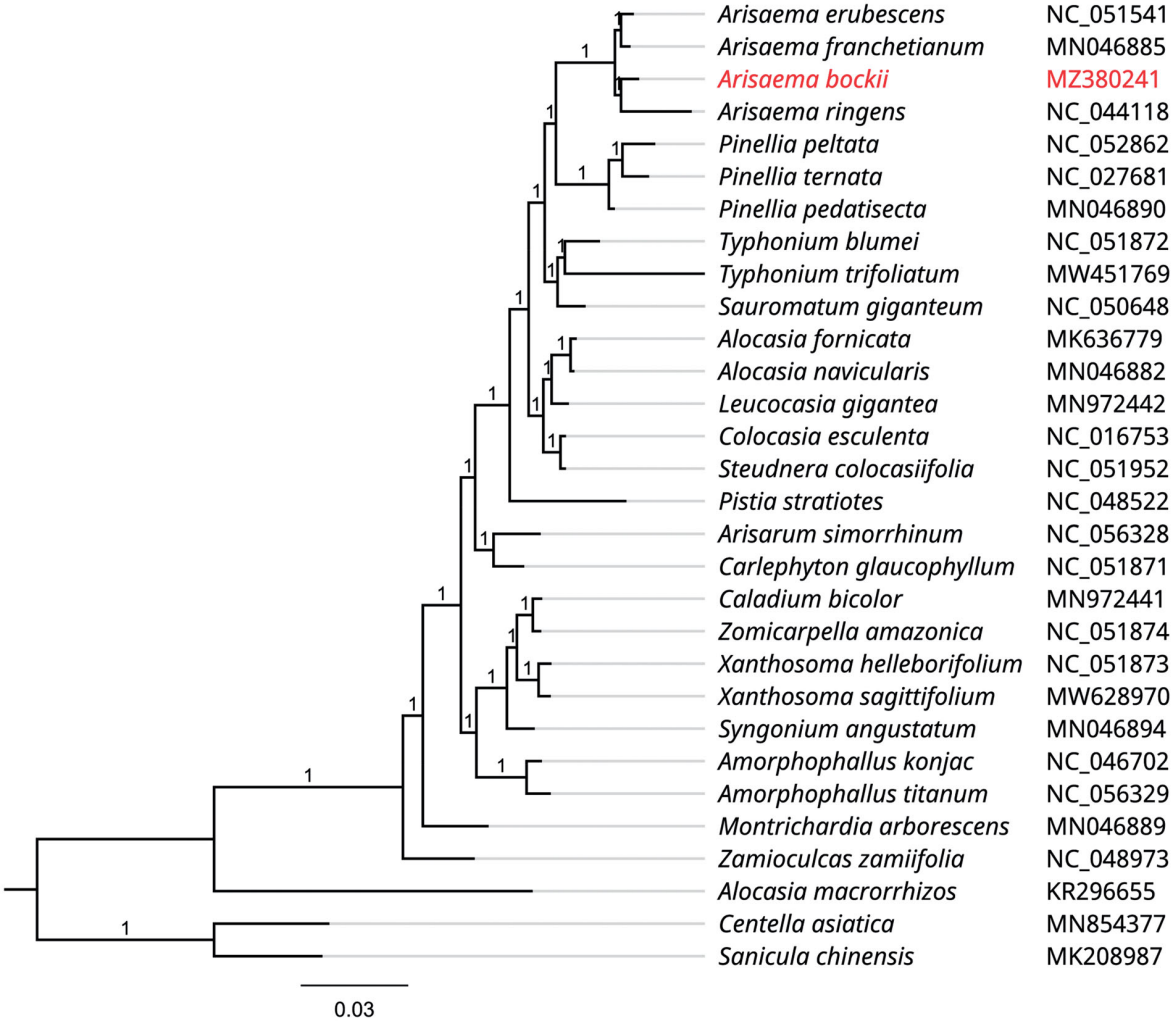
Phylogenetic tree inferred by Maximum Likelihood (ML) method based on 30 representative species. *Centella asiatica* and *Sanicula chinensis* were used as an outgroup. A total of 1000 bootstrap replicates were computed and the bootstrap support values were shown at the branches. GenBank accession numbers were shown in Figure 1.

## Data Availability

The genome sequence data of *A. bockii* that support the findings of this study are openly available in GenBank of NCBI at (https://www.ncbi.nlm.nih.gov/) under the accession no. MZ380241. The associated BioProject, SRA, and Bio-Sample numbers are PRJNA737041, SRR14793494, and SAMN19678498, respectively.
